# MicroRNAs in endometriosis: bioinformatics resources, machine learning strategies, and multi-omics perspectives

**DOI:** 10.1186/s12967-026-08310-y

**Published:** 2026-05-25

**Authors:** Cuishan Guo, Qing Liu, Darong Hai, Xingyu Zhu, Zefei Mo, Yi Wang, Qi Zhao, Chiyuan Zhang

**Affiliations:** 1 Department of Obstetrics and Gynecology, Shengjing Hospital of China Medical University, Shenyang, 110004 China; 2 School of Nursing, Wenzhou Medical University, Wenzhou, 325000 China; 3 The First School of Medicine, School of Information and Engineering, Wenzhou Medical University, Wenzhou, 325000 China; 4 School of Biomedical Engineering, School of Ophthalmology and Optometry, Eye Hospital, Wenzhou Medical University, Wenzhou, 325000 China; 5 School of Computer Science and Software Engineering, University of Science and Technology Liaoning, Anshan, 114051 China

**Keywords:** Endometriosis, microRNAs, Bioinformatics, Machine learning, Multi-omics integration

## Abstract

**Background:**

Endometriosis is a heterogeneous gynecological disorder characterized by chronic pain, infertility, and substantial impairment of quality of life. Increasing evidence indicates that microRNAs (miRNAs) are key regulators of endometriosis pathogenesis through their effects on inflammation, angiogenesis, cell proliferation, fibrosis, and hormone-responsive pathways.

**Methods:**

In this review, we summarize the biological roles of miRNAs in endometriosis and discuss their emerging value as diagnostic biomarkers and therapeutic targets. We further examine major bioinformatics resources and analytical tools used in miRNA research, including databases, target prediction platforms, and expression profiling approaches, with emphasis on their relevance and limitations in the context of endometriosis. In addition, we review recent advances in machine learning and deep learning for miRNA identification, target prediction, regulatory network reconstruction, and miRNA–disease association modeling. Particular attention is given to multi-omics integration strategies, which may better capture the molecular heterogeneity of endometriosis and improve biologically informed stratification.

**Results:**

This review highlights the key roles of miRNAs in endometriosis-related inflammation, angiogenesis, proliferation, fibrosis, and hormone-responsive signaling, and summarizes their potential as non-invasive biomarkers and therapeutic targets. It also emphasizes the value of bioinformatics, machine learning, and multi-omics approaches in identifying clinically relevant miRNA signatures, while acknowledging current challenges in standardization, validation, and interpretability.

**Conclusions:**

Future studies should prioritize standardized multicenter datasets, explainable artificial intelligence, and integrative multi-omics frameworks to develop robust and clinically applicable miRNA-based diagnostic and therapeutic strategies for endometriosis.

## Introduction

Endometriosis is a chronic, estrogen-dependent inflammatory disease that primarily affects the female reproductive system. Due to its progressive nature and tumor-like biological behaviors, including invasion, adhesion, and recurrence, endometriosis is sometimes described as a benign disease with malignant-like features rather than a benign tumor. The disease is closely associated with estrogen and affects approximately 2% to 10% of reproductive-age women [1]. Endometriosis predominantly affects women of reproductive age, although symptoms may persist beyond this period in some patients, and patients often experience severe dysmenorrhea, chronic pelvic discomfort, infertility, fatigue, anxiety, and depression, all of which significantly impair quality of life and increase the risk of ovarian cancer [2]. In rare extrapelvic cases, endometriotic lesions have been reported in sites such as the lungs, liver, gastrointestinal tract, and, exceptionally, the central nervous system. However, severe organ dysfunction appears uncommon and should be interpreted cautiously in a case-specific clinical context [3]. Current treatment options are limited, and recurrence rates are high, necessitating the development of new, more effective diagnostic and therapeutic strategies to improve treatment outcomes and patient quality of life.

In recent years, miRNAs have emerged as key regulators of gene expression and their involvement in endometriosis has received increasing attention. MiRNAs regulate gene expression by binding to target mRNAs, thereby influencing important biological processes such as cell proliferation, differentiation, and apoptosis [4]. The miRNA expression profiles of endometriosis patients exhibit significant differences compared to healthy women, and these abnormally expressed miRNAs may play a key role in the onset and progression of the disease [5, 6]. Despite the growing recognition of the potential of miRNAs in endometriosis, there is currently a lack of effective miRNA biomarkers and therapeutic targets. With the application of advanced computational methods such as machine learning (ML) and deep learning (DL), the accuracy and efficiency of miRNA research have been significantly improved. These advances provide new opportunities to leverage miRNAs for precision diagnosis and personalized treatment of endometriosis.

This review summarizes the potential functions of miRNAs in endometriosis, analyzes how they influence disease progression by regulating key molecular pathways (such as inflammation and cell proliferation), introduces commonly used bioinformatics tools and databases, and discusses the application of ML methods to enhance the accuracy of miRNA research. Finally, this review discusses current challenges in miRNA research and explores the potential applications of miRNAs in early diagnosis, precision treatment, and personalized medicine. This review may provide useful insights for clinical decision-making in endometriosis. Unlike previous reviews that mainly focus on miRNA dysregulation or individual biomarker candidates, this review integrates biological mechanisms, bioinformatics resources, ML/DL-based prediction models, and multi-omics strategies to provide a more computationally informed and translational perspective on miRNA research in endometriosis.

## The association between miRNA and endometriosis

### Basic concepts and biological functions of miRNA

MiRNAs are small non-coding RNA molecules, typically 21–24 nucleotides in length, that were first discovered in 1993 and have since been shown to play important roles in gene expression regulation [7]. MiRNA regulates important biological processes such as development, differentiation, proliferation, and apoptosis by binding to target mRNA, leading to mRNA degradation or translation inhibition [8]. Additionally, miRNAs facilitate intercellular communication via extracellular vesicles such as exosomes, influencing the behavior of neighboring cells [9]. Dysregulation of miRNAs is associated with various diseases, including cancer, cardiovascular diseases, and metabolic disorders, highlighting their potential as biomarkers and therapeutic targets [10].

**Fig. 1:**
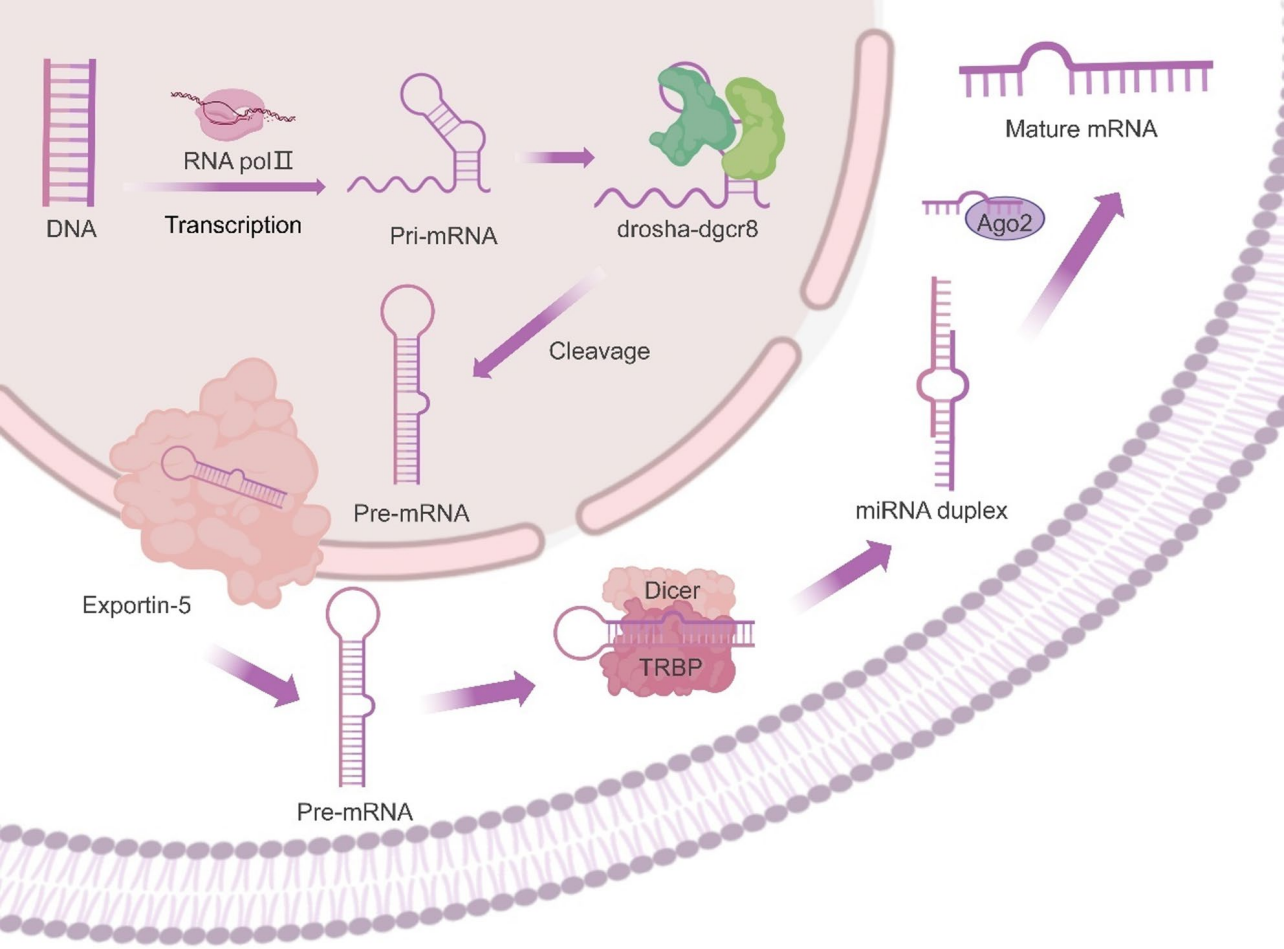
Biogenesis and maturation of miRNAs. The intricate process of miRNA biogenesis initiates within the nucleus, where primary miRNA (pri-miRNA) transcripts are synthesized from DNA via RNA polymerase II. Subsequently, these pri-miRNAs undergo processing by the microprocessor complex, which comprises the enzyme Drosha along with its cofactor DGCR8, facilitating their transformation into precursor miRNAs (pre-miRNAs). Then, pre-miRNAs are shuttled from the nucleus to the cytoplasm, the enzyme Dicer further cleaves the pre-miRNAs into mature miRNA duplexes. From this duplex structure, one strand becomes integrated into the RNA-induced silencing complex, while the complementary strand is typically subjected to degradation

The biogenesis of miRNAs is a complex process, involving transcription from DNA into primary miRNAs (pri-miRNAs), processing by the Drosha/DGCR8 complex into precursor miRNAs (pre-miRNAs), and further processing by Dicer to generate mature miRNAs [11] (Fig. 1). This process is crucial for maintaining normal cellular function, and its dysregulation may contribute to the development of tumors and neurodegenerative diseases [12]. Studies have also shown that splicing factors and translation factors involved in the miRNA biogenesis process are closely associated with miRNA stability and function [13, 14].

### Diagnostic and therapeutic challenges in endometriosis

**Fig. 2:**
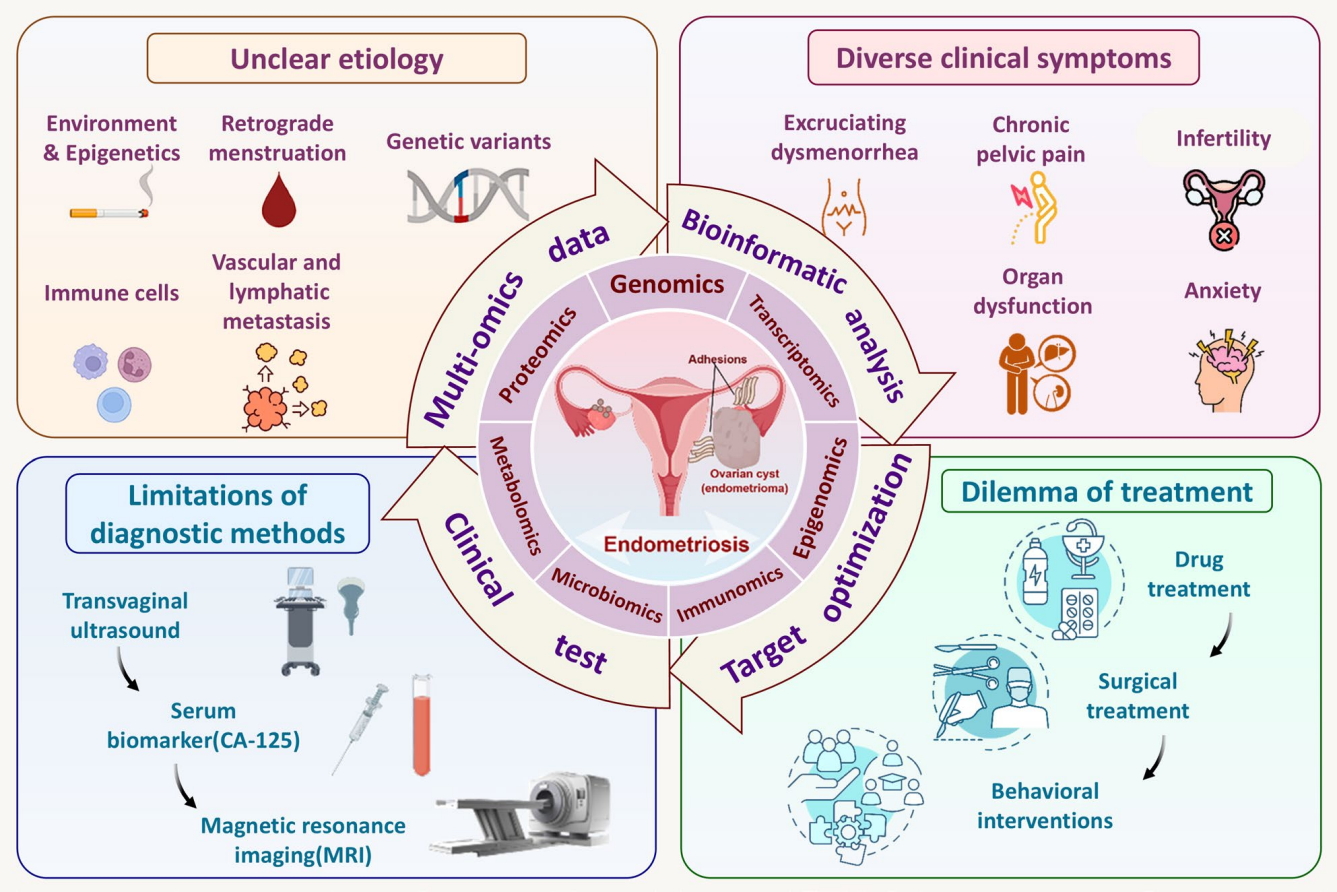
Dilemma of diagnosis and treatment of endometriosis. The etiology of endometriosis remains unclear, and its complex pathogenesis results in a wide range of clinical symptoms, such as different types of pain, infertility, and systemic issues. Also, the options for early diagnosis are very limited, which often leads to delays in both diagnosis and treatment. Additionally, existing treatment methods come with various drawbacks. To address these challenges, it is crucial to integrate genomic, transcriptomic, proteomic, and other omics data, along with bioinformatics analysis, to identify new biomarkers and therapeutic targets for endometriosis

The diagnosis of endometriosis remains challenging. Although laparoscopy is considered the gold standard, its invasiveness and cost limitations prevent many patients from receiving timely diagnosis [15]. Existing non-invasive detection methods, such as transvaginal ultrasound and CA-125 serum markers, lack sufficient specificity and sensitivity, particularly in the early stages [16]. Additionally, the use of imaging techniques like MRI is limited by funding and accessibility [17, 18]. Therefore, there is an urgent need to develop more efficient and accurate early diagnostic tools (Fig. 2).

Therapeutic management of endometriosis remains challenging because its etiology is incompletely understood and its clinical manifestations are heterogeneous. Pharmacological treatments, such as GnRH agonists and progestins, are commonly used to relieve pain and suppress disease activity. However, their long-term use may be limited by side effects and symptom recurrence [19]. Surgical treatment can improve fertility outcomes and remove visible lesions, but postoperative pain recurrence and pelvic adhesions remain important limitations [20]. Emerging strategies, including epigenetic modulators and microbiotherapy, may provide new therapeutic opportunities, but they are still largely at the preclinical stage and continue to face challenges related to targeted delivery, efficacy, and safety [21, 22].

### Potential role of miRNAs in endometriosis

In recent years, research on miRNAs in endometriosis has attracted widespread attention, with multiple potential roles. Compared with healthy women, the miRNA expression profiles of endometriosis patients exhibit significant differences. For example, miRNAs such as miR-155, miR-574-3p, and miR-139-3p have been shown to be dysregulated in multiple studies, revealing their key roles in disease pathology [23]. Specific miRNAs, such as miR-21-5p and miR-34-3p, are closely associated with important pathways such as inflammation, cell proliferation, and apoptosis, promoting the onset and progression of endometriosis [24]. Additionally, abnormal miRNA expression disrupts signaling pathways such as the Wnt/β-catenin pathway, impairing normal cell function and promoting the proliferation and survival of diseased tissues [25].

miRNAs are also considered potential diagnostic biomarkers. For example, serum concentrations of miR-125b-5p, miR-150-5p, and miR-342-3p are significantly elevated in patients with endometriosis, while miR-3613-5p and let-7b are reduced, and these changes can be used to distinguish endometriosis from other gynecological diseases [26]. Some miRNAs, such as miR-17-5p and miR-20a, show significant downregulation in plasma, suggesting their potential as non-invasive diagnostic tools [27].

Abnormal expression of miRNAs in endometriosis is not only associated with disease severity and clinical symptoms, such as pelvic pain and infertility, but also reflects their potential in treatment. For example, miRNAs such as miR-200b, miR-15a-5p, and miR-19b-1-5p have been found to promote angiogenesis through the vascular endothelial growth factor (VEGF) pathway, which is crucial for maintaining diseased tissues [28]. Additionally, the association of miR-135a/b with hormonal imbalances further highlights the potential of miRNAs as therapeutic targets [29].

In summary, the altered expression of miRNAs in endometriosis has improved our understanding of disease pathophysiology and generated a large number of candidate diagnostic biomarkers and therapeutic targets. However, the translation of these candidates into clinical practice has remained limited. A major reason is that endometriosis is biologically heterogeneous across lesion sites, menstrual-cycle phases, symptom profiles, and infertility status, meaning that a biomarker panel performing well in one cohort may not generalize to another. In addition, many published studies are based on relatively small, retrospectively collected datasets with inconsistent sampling procedures and analytical pipelines, making direct comparison and external validation difficult [30]. Therefore, while ML and DL have substantially improved miRNA-disease association prediction, their clinical value will depend less on achieving high in silico AUC values and more on whether they can identify robust, reproducible, and biologically interpretable biomarkers across diverse patient populations. To translate these biological insights into testable hypotheses and clinically meaningful miRNA candidates, appropriate bioinformatics resources and analytical tools are required for data mining, target prediction, functional annotation, and regulatory network construction.

## Application of bioinformatics analysis in miRNA research

### Endometriosis-related databases

Databases are essential resources for miRNA research in endometriosis. Through these databases, researchers can access a wealth of miRNA-related information, including miRNA sequences, expression data, target gene predictions, and functional annotations. These data not only help reveal the potential role of miRNAs in endometriosis but also provide strong support for elucidating the molecular mechanisms of the disease, enabling early diagnosis, improving prognosis assessment, and identifying therapeutic targets. A summary of miRNA databases is provided in Table 1.

**Table 1:** MiRNA database associated with endometriosis

Database	Description	Application example
miRBase	Provides comprehensive miRNA sequence information	Studies the relationship between miRNA and various diseases (such as endometriosis)
miRTarBase	Focuses on miRNA target genes and their interactions	Understands how miRNA affects gene expression related to endometriosis
Endometriosis miRNA	Specifically collects miRNA information related to endometriosis	Aids in the diagnosis and treatment research of endometriosis
ArrayExpress	Offers extensive gene expression data, including miRNAs	Analyzes gene expression changes associated with endometriosis
ENCODE	Focuses on multidimensional data of genomic functions	Studies how gene regulation affects the development of endometriosis
GeneCards	Integrates information on gene function and disease associations	Conducts disease association research on genes related to endometriosis

#### miRBase

miRBase is one of the most widely used miRNA databases globally, providing comprehensive data on miRNAs, including sequences, naming conventions, family relationships, and target gene information [31]. In the study of endometriosis, miRBase provides researchers with basic information about miRNAs and supports miRNA expression difference analysis [32]. Through miRBase, researchers can screen for miRNAs associated with endometriosis and further predict target genes. For example, miRNAs such as miR-181b and miR-29a have been confirmed to be closely associated with endometriosis.

#### miRTarBase

miRTarBase is a specialized database that collects experimentally validated miRNA target genes, providing the number of experimentally validated interactions between miRNAs and target genes [33]. Unlike other prediction databases, miRTarBase provides validated actual data, which is of great significance for enhancing the reliability of miRNA research. In miRNA studies on endometriosis, miRTarBase can be used to validate the actual relationships between different miRNAs and their target genes, helping researchers confirm their roles in the disease. For example, the study of miR-155 in endometriosis has been supported by the miRTarBase database.

#### ArrayExpress

ArrayExpress is a public database storing high-throughput gene expression data, covering various types of gene expression data, including miRNA expression profiles [34]. In miRNA research on endometriosis, ArrayExpress provides a large amount of miRNA expression data from different patient groups, enabling researchers to conduct differential expression analysis of miRNAs and explore the relationship between miRNAs and endometriosis. However, practical multi-omics studies often face challenges such as missing values, incomplete molecular profiles, and unequal sample sizes across genomic, transcriptomic, epigenomic, and clinical data layers, which may affect model training, feature integration, and external validation.

#### ENCODE

ENCODE is a comprehensive resource for genome annotation data, providing molecular-level data such as transcriptomes, epigenomes, and non-coding RNAs (e.g., miRNAs) [35].

In the study of endometriosis, ENCODE provides researchers with additional epigenetic information about miRNAs, revealing their role in the disease and their regulatory mechanisms. Using ENCODE data, researchers can investigate the interactions between miRNAs and other genomic regions, as well as their potential pathogenic roles in endometriosis.

#### GeneCards

GeneCards is a comprehensive gene database that contains detailed information about genes, miRNAs, and related proteins [36]. In miRNA research related to endometriosis, GeneCards provides researchers with information on miRNAs associated with endometriosis and their functions. Through GeneCards, researchers can further analyze the expression patterns of miRNAs in endometriosis and their potential functions, thereby elucidating their role in the onset and progression of the disease.

### Bioinformatics tools and techniques

#### MiRNA target prediction tools

MiRNA target prediction is a critical step in elucidating regulatory mechanisms in endometriosis, but the interpretation of predicted targets requires caution. Most widely used prediction tools are developed as general-purpose resources and not trained specifically on endometriotic tissues, endometrial stromal cells, or lesion-specific molecular environments. As a result, their predictions are often driven by sequence-level rules, such as seed matching, thermodynamic stability, and 3′UTR conservation, while giving insufficient weight to endometriosis-relevant factors including non-canonical binding, alternative 3′UTR usage, tissue-specific isoforms, RNA-binding protein competition, inflammatory activation, fibrosis, and hormone-dependent transcriptional states. Therefore, these tools remain useful for hypothesis generation, but their outputs should not be interpreted as disease-specific evidence unless supported by endometriosis-matched expression data and experimental validation. Several predictive tools are listed in Table 2.

**Table 2:** Target prediction tools for miRNAs

miRNA target prediction tool	Description	Link
TargetScan	Predicts miRNA targets based on conservatism, suitable for screening key regulatory genes for endometriosis.	https://www.targetscan.org/vert_80/
miRanda	Prediction of non-conserved sites, suitable for initial screening of candidate target genes and revealing disease-related pathways	http://mirtoolsgallery.tech/mirtoolsgallery/node/1055
miRDB	Predict targets based on machine learning, supporting functional annotation and pathway enrichment analysis	https://mirdb.org/
miRWalk	Integrate the results of multiple algorithms and support miRNA-mRNA co-expression network construction	http://mirwalk.umm.uni-heidelberg.de/
miRcode	Focus on lncRNA and coding gene target prediction to support inflammation and fibrosis related research	http://www.mircode.org/mircode/browse.php
miRGator	Integrate expression profiling and function prediction to hypothesize the biological roles of miRNAs in diseases	http://mirgator.kobic.re.kr/
DIANA-microT-CDS	Predicts miRNA interactions with CDS/3’UTR to support target confidence scoring and immunoregulatory analysis	http://mirtoolsgallery.tech/mirtoolsgallery/node/1084

##### TargetScan

TargetScan is an important tool for predicting miRNA target genes, capable of identifying potential target genes based on miRNA sequence information and providing details such as binding sites and conservation [37]. In the study of endometriosis, TargetScan helped researchers identify miRNA target genes and reveal the regulatory role of miRNAs in this disease. By combining TargetScan data, researchers were able to more accurately screen for potential pathogenic miRNAs and their target genes, further elucidating their molecular mechanisms in endometriosis.

##### miRanda

miRanda is an earlier prediction tool that evaluates miRNA–mRNA interactions using sequence complementarity and binding energy, and it is often considered more permissive than conservation-driven tools. This feature makes miRanda potentially useful for capturing candidate non-conserved or non-canonical binding events that may be missed by stricter seed-based predictors. Nevertheless, this broader search space comes at the cost of increased false-positive predictions, especially when tissue-specific transcript architecture and cellular context are not incorporated. In endometriosis, this is particularly relevant because ectopic lesions and endometrial stromal cells may display disease-specific transcript isoforms and microenvironment-dependent regulatory states that cannot be resolved by sequence matching alone.

##### miRDB

miRDB uses ML (MirTarget algorithm) to predict target genes, covering the 5’UTR, coding regions (CDS), and 3’UTR regions. It provides functional annotations and pathway enrichment results for target genes [38]. Combining miRDB with TargetScan results can improve the accuracy of target prediction in endometriosis research.

##### miRWalk

miRWalk integrates the prediction results from the previous tools to provide a more comprehensive list of target genes [39]. By combining differentially expressed miRNA and mRNA data, it constructs a miRNA-mRNA co-expression network, enabling a more comprehensive study of the disease.

##### miRcode

miRcode is a graph-based tool based on GENCODE gene annotations that predicts miRNA targets in the human transcriptome, including lncRNAs and coding genes. Users can input gene symbols or access sites, select different screening criteria, view results, and download data. In endometriosis research, it can predict miRNA targets regulating inflammatory factors (e.g., TNF-α, IL-6) or fibrosis-related genes (e.g., TGF-β1).

##### miRGator

The miRGator database is a tool for guiding the functional interpretation of miRNAs [40]. Functional analysis and expression profiling combined with target gene prediction enable the inference of the biological functions of miRNAs.

##### DIANA-microT-CDS

DIANA-microT-CDS integrates ML with experimental data to predict interactions between miRNAs and CDS and 3’UTRs, providing target reliability scores. In endometriosis research, it identifies targets regulating immune cell polarization (e.g., macrophages), such as miR-155 targeting SOCS1 to influence inflammatory responses.

#### Expression profiling analysis tools

Expression profiling analysis tools provide a systematic framework for elucidating the regulatory role of miRNAs in endometriosis, from differential expression identification, target gene prediction to functional validation, thereby advancing disease mechanism research and clinical translation. Specific tools are listed in Table 3.

**Table 3:** Expression profiling tools for miRNAs

Analysis tools	Description	Link
GEOexplorer	Visualization and analysis tool for GEO data, suitable for users with no programming knowledge	https://www.bioconductor.org/packages/release/bioc/html/GEOexplorer.html
edgeR	Differential expression analysis for small sample RNA-Seq data, support complex design	https://bioconductor.org/packages/release/bioc/html/edgeR.html
miRDeep2	Focus on miRNA prediction and quantification, suitable for small RNA sequencing data	https://anaconda.org/bioconda/mirdeep2
miRGen	Integrates miRNA target gene and tissue-specific expression information	http://www.microrna.gr/mirgen
DIANA-miRPath v3.0	Predicts miRNA targets in the CDS region to support mechanistic studies	http://www.microrna.gr/miRPathv3
miRanalyzer	All-in-one small RNA analysis platform covering identification and enrichment	http://bioinfo2.ugr.es/miRanalyzer/standalone.html
Chimera	For identification of fusion transcripts, commonly used in cancer research	http://www.ebi.ac.uk/research/enright/software/chimira
MMIA	Supports miRNA and mRNA co-expression network analysis, suitable for multi-omics integration	https://www.hsls.pitt.edu/obrc/index.php/index.php?page=URL1250873924
miTalos	Focused miRNA pathway analysis with target gene network and enrichment	https://mirtoolsgallery.tech/mirtoolsgallery/node/1506

##### GEOexplorer

GEOexplorer is an online analysis tool based on the Gene Expression Omnibus(GEO) database [41], designed to help researchers efficiently mine gene expression data without programming knowledge. The tool integrates functions such as data retrieval, preprocessing, differential analysis, functional enrichment, and visualization, supports microarray and RNA-seq data, and provides interactive chart generation capabilities. It allows users to access, integrate, and analyze gene expression datasets without advanced programming skills, including miRNA expression profiles in endometriosis. The tool supports uploading custom datasets, enabling researchers to validate findings by combining public data with clinical samples.

##### edgeR

edgeR is a tool for differential expression analysis of RNA-Seq data, similar to DESeg2, primarily used for small RNA expression profiling [42]. Through statistical models, edgeR can effectively identify miRNA expression differences under different conditions. In the study of endometriosis, edgeR can help researchers screen for miRNAs with significant expression differences and further analyze their relationship with the disease. Since edgeR can handle large-scale data, it is very practical for high-throughput miRNA expression data analysis.

##### miRDeep2

miRDeep2 is a specialized tool for analyzing small RNA sequences, capable of accurately predicting miRNA expression profiles from high-throughput small RNA-seq data. It not only identifies known miRNAs but also discovers new miRNA molecules, making it particularly suitable for identifying low-expression and rare miRNAs. In the miRNA expression profile analysis of endometriosis, miRDeep2 helped researchers identify potential disease-related miRNAs, providing data support for further research on their role in the disease.

##### miRGen

miRGen v4 [43], released in 2020, enhances the understanding of transcriptional regulation of miRNA biogenesis. However, this was achieved in a different manner by integrating the analysis results of over 1,000 gene expression cap analysis (CAGE) samples from 133 tissues, cell lines, and primary cells, and ultimately providing cell type-specific transcription start sites (TSS) for over 1,500 miRNAs. The dataset was expanded to include ChIP-Seq and DNase-Seq datasets from the miRNA database ENCODE repository mentioned earlier. This is particularly useful for analyzing miRNA expression profiles associated with diseases.

##### DIANA-miRPath v3.0

DIANA-miRPath v3.0 evaluates the regulatory roles of miRNAs and analyzes their pathways, supporting all analyses of KEGG molecular pathways and gene families from seven species [44]. Provides a target gene database (TarBase) with experimental support, ensuring high reliability of results. After completing expression profiling analysis (e.g., obtaining a list of differentially expressed miRNAs), use DIANA-miRPath for functional enrichment to clarify the regulatory network of miRNAs. Can reveal the synergistic regulatory mechanisms of miRNAs in endometriosis.

##### miRanalyzer

miRanalyzer supports standardized processing of small RNA sequencing (sRNA-seq) or chip data, providing miRNA annotation functionality. It is suitable for processing miRNA annotation, differential screening, and target gene prediction in sequencing data [45]. Match known miRNA databases to identify differentially expressed miRNAs between endometriosis patients and healthy controls, different subtypes, or tissue types. Integrate target gene prediction tools (e.g., TargetScan, miRanda) to screen potential target genes and perform GO/KEGG enrichment analysis.

##### Chimera

Chimera supports multi-omics data integration analysis [46]. In endometriosis research, it can integrate multi-omics data and perform functional analysis, elucidating the association between abnormal gene expression and epigenetic regulation in EMS. For example, by integrating RNA-seq data from ectopic endometrium of EMS patients with methylation array data, genes driven by differential methylation can be identified.

##### MMIA

MMIA is a multi-omics integration analysis tool that supports joint analysis of miRNA and mRNA expression profiles [47]. It integrates miRNA-mRNA regulatory networks to reveal the core molecular mechanisms of endometriosis, while also aiding in the development of diagnostic biomarkers, identifying core regulatory networks driving EMS progression, and elucidating epigenetic regulation.

##### miTalos

miTalos performs miRNA target gene enrichment analysis and identifies potential pathway regulation by assessing the functional roles of miRNAs in biological pathways through their network proximity using proximity scores [48]. It can identify core miRNAs driving the progression of endometriosis and elucidate the association between miRNAs and clinical phenotypes.

### Application of computational models in endometriosis

#### Data fusion paradigm

Endometriosis is a biologically heterogeneous disorder in which lesion location, menstrual-cycle phase, infertility status, inflammatory activity, and fibrosis burden all influence molecular readouts [49]. For this reason, single-omics studies often capture only one aspect of disease biology and may fail to explain why apparently similar clinical phenotypes arise from different molecular mechanisms. Multi-omics data fusion is therefore relevant not merely because it improves classification performance, but because it can connect miRNA dysregulation with upstream epigenetic control and downstream transcriptional or protein-level consequences. In the context of endometriosis, the major value of fusion models lies in identifying cross-omics regulatory modules linked to progesterone resistance, immune dysregulation, angiogenesis, extracellular-matrix remodeling, and lesion persistence [50].

Since 2009, MDA prediction models have continued to evolve, with the core modeling path gradually developing from early shallow models that relied on single network topology features to DL systems that integrate multi-source heterogeneous data. Based on modeling strategies and the degree of data integration, MDA models can be broadly categorized into three stages: the initial exploration stage (2009–2012), the structural enhancement stage (2013–2017), and the algorithm optimization stage (2018 to present) [51]. In the first stage, representative models such as the hypergeometric distribution-based scoring method achieved an AUC prediction performance of approximately 0.75 by propagating probabilities in the functional similarity network between miRNAs and diseases [52]. In the second stage, the model structure was expanded by introducing multimodal fusion strategies. For example, the RBMMMDA model integrated miRNA family information, semantic word vectors, and target gene data, and achieved nonlinear combination of multi-source features through a neural network architecture [53]. In the third stage, several advanced algorithms were introduced into the model design, including matrix decomposition (e.g., M2LFL), graph convolutional networks (e.g., MMGCN), tensor decomposition (e.g., TDRC), ensemble learning (e.g., EGBMMDA), and deep neural networks (e.g., MDA-CNN). These methods not only enhance the modeling capability of heterogeneous information but also significantly improve prediction performance, achieving cross-validation AUC values of 0.97 and 0.93 on the HMDD v2.0 and v3.0 datasets, respectively [54]. These studies have confirmed that the optimization of fusion strategies and the ability to express multimodal information have become key factors determining the performance of MDA prediction models.

In endometriosis research, the value of data fusion is not merely higher predictive accuracy, but its ability to connect distinct molecular layers to specific pathological programs. Transcriptomic miRNA changes can reflect inflammatory activation, aberrant stromal proliferation, and angiogenic signaling, whereas epigenomic features help explain more stable lesion-associated states such as progesterone resistance, persistent fibroproliferative remodeling, and epithelial–mesenchymal transition (EMT)-like plasticity. From this perspective, feature-level and multi-omics fusion frameworks are particularly relevant because they enable the joint modeling of miRNAs, their target genes, and upstream regulatory signals within a shared representational space. Rather than functioning only as classifiers, these models can prioritize cross-omics modules that map onto core endometriosis biology, including immune dysregulation, extracellular matrix deposition, Wnt/β-catenin-associated remodeling, and lesion vascularization. Importantly, such frameworks do not by themselves prove causality. Instead, their mechanistic value lies in identifying biologically coherent hypotheses that can subsequently be validated in eutopic and ectopic endometrial tissues.

MiRNA plays a key regulatory role in the occurrence and development of endometriosis, and its expression is influenced by various factors such as DNA methylation, transcription factor activity, and chromatin conformation [55]. Single-omics data alone cannot fully reveal the causal relationship between miRNA and disease. Through multi-omics integration, information from different levels can be integrated to enhance the predictive ability of the association between miRNAs and endometriosis [56]. Genomic data provide genetic variations associated with disease susceptibility (e.g., SNPs, CNVs), transcriptomic data reflect the dynamic expression of miRNAs and their target genes, and epigenomic data reveal the activity status of miRNA gene promoter regions [57]. By integrating these insights, researchers can more accurately identify the regulatory sources of miRNAs and clarify their functional roles in diseases. Researchers have developed various integrated methods to achieve this goal, such as the MOADLN model, which enhances miRNA recognition through self-attention mechanisms [58], the D-miRT model, which combines sequence and epigenetic features to accurately predict miRNA TSS [59], and the Sparse Autoencoder and Multi-Layer Perceptron (SPALP) model, which integrates sparse autoencoders and functional similarity to perform excellently in MDA prediction [60].

Therefore, integrating genomic, transcriptomic, and epigenomic data is an important strategy to improve the accuracy of miRNA-endometriosis association prediction. In the future, by combining multi-omics DL techniques, we aim to construct more interpretable and generalizable MDA models, providing theoretical basis and technical support for the precise diagnosis and personalized treatment of endometriosis.

#### Machine learning models and practical applications

In endometriosis, the significance of ML and DL models should be evaluated not only by predictive performance but also by how well their mathematical structure matches disease biology. Endometriotic lesions are highly heterogeneous across patients, lesion sites, and menstrual-cycle states, and they involve interacting processes such as inflammation, angiogenesis, fibrosis, hormone-response dysregulation, and EMT-like remodeling [61]. Accordingly, different model families offer different mechanistic advantages [62]. CNN-based methods are useful for extracting local sequence motifs relevant to miRNA maturation and binding, recurrent architectures are better suited to capturing dynamic and state-dependent regulation, whereas graph-based models are more informative for reconstructing the relational structure among miRNAs, target genes, and disease phenotypes [63, 64]. Therefore, a biologically meaningful review of these models should ask not only which method performs best, but also which pathological process of endometriosis each architecture is best positioned to resolve [65]. The listed ML models are shown in Table 4.

**Table 4:** Application of miRNA-related models in endometriosis

Function category	Model	Application in endometriosis
miRNA target prediction & identification	BiLSTM + word2vec	Improves miRNA target prediction accuracy
	CNN/RNN	Identifies miRNA targets and sequences
	Random forest	Predicts miRNA targets from multi-dimensional features
miRNA prediction & functional analysis	Transformer architecture	miRe2e model for miRNA analysis in tumors and endometriosis
	GRU-GCN model	Models miRNA regulatory networks in endometriosis
	Deep neural network	Learns complex features for accurate miRNA prediction
miRNA regulation & expression studies	D-miRT model	Improves miRNA TSS prediction accuracy
	SPALP model	Predicts miRNA-disease associations
	Autoencoder-based model	Extracts features for miRNA expression and regulation studies
miRNA-lncRNA interaction studies	PmliPred model	Predicts miRNA-lncRNA interactions in endometriosis
	miRNA-lncRNA co-expression analysis	Identifies functional relationships between miRNA and lncRNA
	GNN	Models miRNA-lncRNA interactions and regulatory networks
miRNA subcellular localization studies	RNALoc-LM	Predicts miRNA subcellular localization
	Seq2Loc model	Predicts RNA subcellular localization
	LSTM + attention Mechanism	Enhances localization prediction with attention mechanism
miRNA-RBP interaction studies	DeepMiRBP model	Predicts miRNA-RBP interactions in endometriosis
	MiRTar	Combines data for miRNA-RBP interaction recognition
	BindingDB	Provides RBP-miRNA interaction data
Multi-omics data integration	DeepOmics model	Integrates multi-omics data for miRNA interaction analysis
	Multi-omics fusion model	Combines omics data for accurate miRNA prediction
	iCluster	Integrates multi-omics data for disease subtyping
Single-cell data analysis	SCA model	Analyzes single-cell miRNA expression profiles in endometriosis
	SingleCellNet model	Predicts miRNA-disease associations in single-cell data
	Seurat	Identifies miRNA functional differences across cell types

##### Transformer architecture revolutionizes miRNA prediction workflow

The mechanistic relevance of Transformer-based models such as miRe2e lies not simply in end-to-end prediction, but in their ability to capture long-range dependencies across miRNA precursor sequences without relying on handcrafted features [66]. This property is especially meaningful for endometriosis because lesion-associated miRNAs are unlikely to act through isolated local motifs alone; instead, their maturation and abundance may depend on distributed sequence contexts that ultimately affect inflammatory signaling, angiogenesis, and hormone-responsive pathways [67]. In this sense, global attention offers a conceptual advantage for prioritizing miRNAs whose downstream targets converge on processes already implicated in endometriosis, such as Wnt/β-catenin-associated remodeling, EMT-like transitions, and progesterone-response abnormalities. However, the biological contribution of miRe2e should be interpreted cautiously. The model can prioritize candidate regulatory miRNAs, but mechanistic conclusions still require integration with target-gene, pathway-enrichment, and tissue-expression evidence [68].

##### Multimodal fusion models enhance the accuracy of TSS prediction

The prediction of miRNA TSS is crucial for understanding their expression regulatory mechanisms. Traditional RNA-Seq struggles to resolve their precise locations [69]. The D-miRT model, based on a dual-stream convolutional network, integrates sequence features with epigenetic information (such as DNase-Seq and histone modifications), significantly improving the prediction accuracy of miRNA TSS [59].

The application of this model helps reveal the upstream regulatory structure of miRNA expression in endometrial heterotopic tissue, promoting the construction of disease heterotopic expression profiles. Additionally, other researchers have developed an alternative model, SPALP, which combines a sparse autoencoder with a multilayer perceptron (MLP). This model integrates miRNA functional similarity and disease semantic information to effectively compress high-dimensional features. It achieved an AUC of 0.9859 and an accuracy rate of 94.61% across multiple datasets [60], making it suitable for predicting and screening unknown MDA.

##### GRU-GCN model combining graph structure and temporal dynamics

The appeal of GRU-GCN architecture in endometriosis is that the disease is both network-driven and temporally dynamic. The GCN component is well suited to modeling the relational structure among miRNAs, target genes, and disease phenotypes, which is relevant for reconstructing inflammatory, angiogenic, and fibrosis-associated regulatory circuits [70]. The GRU component adds biological value by capturing state transitions over time, an important feature in a hormone-responsive disorder characterized by menstrual-cycle-dependent fluctuations and persistent progesterone resistance. From a mechanistic perspective, this makes GRU-GCN more informative than static classifiers for studying how regulatory interactions may shift from eutopic endometrium to ectopic lesions, or from early lesion establishment to chronic fibrotic remodeling. Nevertheless, its current role should be framed as hypothesis generation rather than proof of temporal causality, because predicted dynamic associations still require longitudinal or stage-specific experimental validation [67].

##### Deep encoding strategy for predicting miRNA-lncRNA interactions

Furthermore, in modeling the interaction between miRNAs and lncRNAs, the PmliPred model combines DL methods with traditional ML methods. By training on the raw features of miRNA and long non-coding RNA (lncRNA) sequences as well as artificially engineered features, it achieves robust predictive performance. The PmliGKKS model employs advanced two-dimensional Kmer cross-coding technology to merge miRNA and lncRNA features into coherent encoded images, thereby facilitating interactive prediction [71, 72]. This pioneering approach synergizes neural networks capable of processing sequence and image data, significantly enhancing the ability to extract features related to RNA sequences and their structures. Empirical evidence demonstrates that PmliGKKS exhibits high generalization ability across different species, providing a technical foundation for high-throughput screening of miRNA-lncRNA regulatory axes associated with endometriosis.

##### Deep characterization modeling of miRNA subcellular localization

miRNA subcellular localization is closely associated with its regulatory functions [73]. RNALoc-LM is a DL framework based on a pre-trained RNA language model, which excels at capturing inherent local patterns and long-range dependencies within sequences. By integrating TextCNN and BiLSTM modules, RNALoc-LM can effectively identify key sequence features critical for accurately predicting miRNA subcellular localization [74]. Additionally, the model incorporates a multi-head attention mechanism, enabling it to focus on important regions within RNA sequences, thereby enhancing its expressiveness and prediction accuracy, which is beneficial for elucidating the functional location specificity of miRNAs in endometriosis.

##### Integrating sequence and structural information for miRNA-RBP interaction prediction

RNA-binding proteins (RBPs) play a crucial role in miRNA stability and target regulation. DeepMiRBP, an advanced hybrid DL framework, has been introduced to predict interactions between miRNAs and RBPs. The DeepMiRBP model first employs a Bi-LSTM neural network to effectively capture sequence dependencies and contextual subtleties within RNA sequences, and integrates an attention mechanism to emphasize the most relevant features. Additionally, the model combines a CNN to assess protein structure-related spatial data, generating a comprehensive representation of potential miRNA binding sites derived from position-specific score matrices and contact maps. This unified strategy enables DeepMiRBP to integrate RNA sequence features and protein spatial structural data, significantly enhancing the model’s predictive performance and flexibility, particularly in complex biological frameworks [75]. During training, DeepMiRBP achieved a prediction accuracy of 87.4%, with slightly lower performance on the test dataset at 85.4% and an f-score of 0.86. Researchers conducted three targeted case studies centered on miR-451, miR-19b, miR-23a, miR-21, miR-223, and let-7d, further validating the model’s potential for identifying miRNAs and novel RBPs [75]. This strategy provides a foundation for elucidating the specific miRNA-RBP regulatory mechanisms in endometriosis.

##### Multi-omics deep integration model for identifying miRNA disease associations

MOADLN should be discussed not only as a high-performing classification framework, but also as a potentially informative model for disentangling the layered pathology of endometriosis. Its self-attention mechanism is particularly relevant because endometriotic lesions are likely driven by sparse but biologically meaningful cross-omics couplings, for example between promoter methylation, miRNA dysregulation, and downstream inflammatory or fibrotic gene expression. By weighting these dependencies, MOADLN may help distinguish whether a candidate miRNA is more strongly linked to immune activation, extracellular matrix remodeling, epithelial–stromal plasticity, or hormone-response failure [76]. This is especially important in endometriosis, where similar clinical phenotypes can arise from different molecular routes. Therefore, the mechanistic importance of MOADLN lies less in generic multi-omics classification and more in its capacity to stratify lesions according to dominant pathogenic programs, which could ultimately support biologically informed subtyping and personalized intervention.

##### SCA-based single-cell miRNA modeling

At the single-cell level, the Sparse Connectivity Autoencoder (SCA) provides a dimensionality-reduction and feature-compression framework for miRNA expression modeling, enabling denoising, clustering, and identification of cell type-related miRNA expression patterns. By limiting the number of connections between the input layer and the decoder, SCA enhances model interpretability and effectively suppresses high-dimensional noise, facilitating the identification of potential cell type-related miRNA expression patterns in unsupervised settings. In single-cell RNA sequencing data applications, SCA can accurately capture biologically meaningful miRNA expression features and reveal subtle differences between cell subpopulations [77]. This model supports the integration of heterogeneous single-cell data collected under different experimental conditions, significantly improving cross-experimental comparability and biological interpretation efficiency. In endometriosis research, SCA has the potential to identify miRNA expression profiles closely related to lesion cell characteristics, thereby providing new algorithmic tools and research pathways for constructing miRNA regulatory networks in different pathological subtypes.

With the development of multimodal omics technologies and the continuous accumulation of clinical samples, deep learning-based miRNA prediction models may contribute to endometriosis research by extracting sequence features, reconstructing miRNA-target interaction networks, reducing high-dimensional omics noise, and weighting cross-omics regulatory signals relevant to early diagnosis, classification, and personalized treatment. However, current challenges include model interpretability, data imbalance, and cross-species generalization ability. Future studies should further combine GNN, transfer learning, and pre-training frameworks to construct more robust and interpretable computational models, providing a more solid data foundation for the precision medicine of endometriosis.

## Future research directions and challenges

In this review, we systematically elucidated the important role of miRNAs as biomarkers and potential therapeutic targets in endometriosis and deeply analyzed their application in this field through bioinformatics analysis. At the same time, we also discussed the advantages and disadvantages of current bioinformatics tools and computational models. Based on the latest research results, the following four aspects summarize the future research directions and challenges.

### Standardization of miRNA detection techniques

Currently, one of the major obstacles preventing miRNA biomarkers for endometriosis from entering clinical practice is the lack of standardized datasets and detection workflows [78]. Although many studies report promising diagnostic performance, the underlying samples often differ in specimen type, menstrual-cycle timing, lesion subtype, sequencing platform, normalization method, and case definition [79]. As a result, even biomarkers or models with excellent performance in a single study may fail during external validation and cannot easily support regulatory-grade evidence. For ML-based diagnostics, this problem is particularly critical because model performance is highly dependent on training data quality and consistency. Therefore, future progress requires not only larger datasets, but also prospective, multicenter, and protocol-harmonized cohorts, together with standardized preprocessing and reporting frameworks. Such efforts would allow computational models to learn clinically stable rather than cohort-specific signals, thereby improving the reproducibility and translational potential of miRNA-based diagnostics in endometriosis [80].

### Bioinformatics technological innovation and iteration

With the continuous development of bioinformatics tools, the development of new tools has provided more opportunities for the study of miRNAs in endometriosis. Currently, various specialized web servers play an important role in enrichment analysis and regulatory network construction [51]. In the future, combining ML algorithms with innovative tool functions will be an important development trend. CNNs and RNNs have demonstrated strong feature extraction capabilities, potentially enhancing the accuracy and efficiency of miRNA target gene prediction tools. For example, CNNs can automatically extract features from pre-miRNA sequences, facilitating their classification and corresponding target prediction, thereby eliminating the need for traditional manual feature selection [81]. Furthermore, CNNs and RNNs can effectively capture structural and temporal features in miRNA-target interactions [82] and process miRNA expression data with temporal sequence characteristics. Such technologies provide important support for studying the dynamic changes of miRNAs in different stages of endometriosis and the pathogenesis of the disease [83].

### Deep integration and mining of multi-omics data

Endometriosis is a biologically heterogeneous disease with substantial variation in lesion location, inflammatory status, fibrosis burden, hormonal responsiveness, pain phenotype, and reproductive outcome. This heterogeneity is one of the main reasons why many candidate biomarkers and predictive models, despite reporting high AUC values, have not translated into robust clinical tools. A single omics layer rarely captures the full molecular diversity of the disease, which means that apparently high-performing models may in fact be learning subtype-specific or cohort-specific patterns. Multi-omics integration therefore has value not simply because it improves classification accuracy, but because it may help decompose endometriosis into biologically meaningful molecular programs, such as inflammatory-dominant, fibrosis-dominant, angiogenic, or progesterone-resistant states. In this context, frameworks such as MOADLN are promising because they can model cross-omics dependencies rather than isolated biomarkers. The translational goal should thus shift from identifying a universal marker toward developing stratified and biologically grounded diagnostic models that remain valid across heterogeneous patient groups.

### Optimizing ML algorithms

With the continuous expansion of data volume, ML and especially DL have shown considerable promise in endometriosis research [84]. However, the key bottleneck for clinical translation is no longer simply predictive accuracy. Many models achieve strong in silico performance, yet remain difficult to deploy in clinical settings because their decision logic is opaque, their behavior under distribution shift is unclear, and clinicians cannot easily determine whether a prediction is driven by biologically plausible features or technical artifacts [85]. For this reason, the next generation of computational models should place greater emphasis on explainable artificial intelligence (XAI). In practical terms, XAI methods such as attention visualization, feature attribution, pathway-level explanation, and uncertainty estimation could help identify which miRNAs, regulatory modules, or omics layers are driving model outputs. This would make it easier to distinguish clinically meaningful biomarkers from spurious correlations, improve clinician trust, and support prospective validation. Therefore, future algorithm optimization in endometriosis should not be framed only as improving AUC or accuracy, but as building robust, externally validated, and interpretable models that can satisfy both biological scrutiny and clinical usability [86].

## Conclusion

Overall, the research prospects for miRNA as a biomarker for endometriosis are promising, but its clinical translation requires more specific priorities, including standardized sampling and detection workflows, prospective multicenter validation, and externally reproducible biomarker panels. Future studies should further integrate multi-omics data with interpretable computational models to clarify miRNA regulatory networks, improve biological transparency, and support clinically applicable patient stratification. These efforts may provide a stronger foundation for early diagnosis, prognosis assessment, and personalized treatment of endometriosis.

## Data Availability

Not applicable.

## Abbreviations

ML: Machine learning
DL: Deep learning
pri-miRNAs: Primary miRNAs
pre-miRNAs: Precursor miRNAs
VEGF: Vascular endothelial growth factor
MDA: miRNA-disease association
CDS: Coding regions
CAGE: Gene expression cap analysis
TSS: Transcription start sites
TarBase: Target gene database
sRNA-seq: Small RNA sequencing
MOADLN: Multi-omics attention deep learning networks
Autoencoder: Autoencoder networks
GNN: Graph neural networks
SPALP: Sparse Autoencoder and Multi-Layer Perceptron
SVM: Support vector machine
CNN: Convolutional neural network
RNN: Recurrent neural network
Bi-LSTM: Bidirectional long short-term memory
MLP: Multilayer perceptron
